# A familial chromosomal complex rearrangement confirms RUNX1T1 as a causative gene for intellectual disability and suggests that 1p22.1p21.3 duplication is likely benign

**DOI:** 10.1186/s13039-019-0440-6

**Published:** 2019-06-14

**Authors:** Fabrizia Restaldi, Viola Alesi, Angela Aquilani, Silvia Genovese, Serena Russo, Valentina Coletti, Daniele Pompili, Roberto Falasca, Bruno Dallapiccola, Rossella Capolino, Matteo Luciani, Antonio Novelli

**Affiliations:** Bambino Gesù Children’s Hospital, Rome, Italy

**Keywords:** Complex chromosomal rearrangements, RUNX1T1, 1p22.1p21.3 duplication, 8q21.3q22.1 deletion

## Abstract

**Background:**

Complex chromosomal rearrangements are constitutive structural aberrations involving three or more breaks. They can be balanced or unbalanced and result in different outcomes, depending on deletion/duplication of genomic material, gene disruption, or position effects.

**Case presentation:**

We report on a patient presenting with severe anemia, splenomegaly, mild intellectual disability and facial dysmorphisms harboring a 4.3 Mb duplication at 1p22.1p21.3 and a 2.1 Mb deletion at 8q21.3q22.1, involving RUNX1T1 gene. The healthy brother presented the same duplication of chromosome 1p as at 1p22.1p21.3.

**Conclusions:**

The rearrangement found both these siblings resulted from malsegregation in the proband and recombination in her healthy brother of a balanced paternal complex chromosomal rearrangement. These results confirm RUNX1T1 as a causative gene for intellectual disability and suggest the 1p22.1p21.3 duplication is likely benign.

## Introduction

Complex chromosomal rearrangements (CCRs) are constitutive structural aberrations, which involve three or more chromosomal breaks resulting in exchanges of genetic material. They are rare in human population [1], with an estimated 0.5% occurrence in newborns [2]. They are classified into three groups, basing on rearrangement type and breakpoints number [3]: 1. Three-way exchange with three breaks in three different chromosomes, the most common type of CCRs; they are usually familial and transmitted by the mother. 2. Double two-way exchange with a coincidence of two separate simple reciprocal translocations, often described as double or multiple rearrangements. 3. Exceptional CCRs associated with a more complicated rearrangement; they are mostly de novo and commonly associated with an abnormal phenotype. When unbalanced, they can result in abnormal phenotypes, depending on size and gene content of the affected regions. Also balanced CCR can result in a clinical phenotype as a consequence of gene disruption at the breakpoint or position effect of genes flanking the rearranged regions. Balanced CCRs can also undergo malsegregation during meiotic division, leading to unbalanced derivative chromosomes and affected offspring [4].

In the present study, we report on a patient presenting with severe anemia, splenomegaly, mild intellectual disability and facial dysmorphisms and harboring a double imbalance resulting from malsegregation of a paternal CCR. We also report on her brother presenting with mild facial dysmorphic features, who harbored a different chromosomal rearrangement due to a meiotic recombination of a paternal CCR. Genotype-phenotype association and published data argue for a pathological role for RUNX1T1 gene imbalance, which can be regarded as an interesting candidate gene for intellectual disability.

## Case report

We report on a patient already described by Valli R. et al. [5] providing further data missing in the previous report. In fact, locus specific FISH analysis of patient and first-degree relatives allowed elucidating both familial reproductive risk and genotype-phenotype association.

**Fig. 1:**
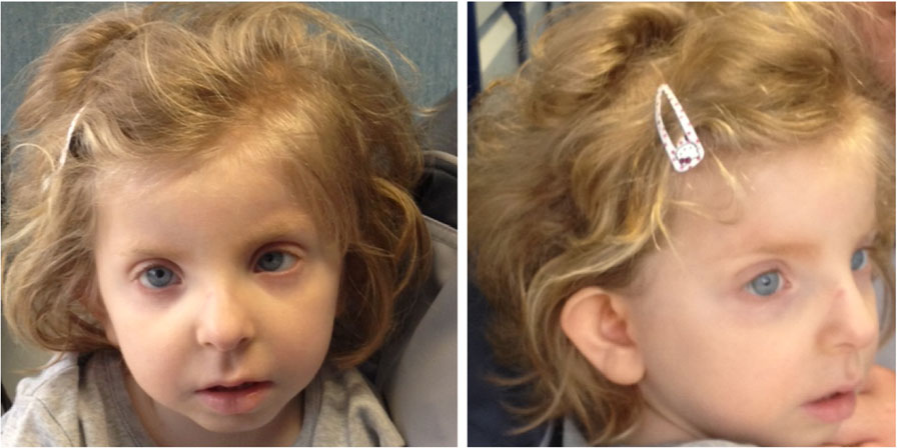
Patient at 3 years old. Note dysmorphic facial features (blepharophimosis, ocular hypertelorism, long philtrum and abnormal ear configuration

This 9 year-old girl, child of non-consanguineous healthy parents, was born at term, with pre- and post-natal growth retardation, a surgically resolved atrial septal defect, dysmorphic facial features including blepharophimosis, ocular hypertelorism, long philtrum and abnormal ear configuration (Fig. 1), severe anemia and seizures. Metabolic diseases were excluded.

Because of persistent severe anemia and distinct clinical features a bone marrow biopsy was carried out which revealed unremarkable morphology of hematopoietic cells but confirmed erythroid hypo-cellularity. Standard karyotype analysis from peripheral blood was normal and FISH analysis searching for chromosome 7 monosomy or chromosome 8 trisomy on bone marrow cells tested negative. Fanconi Anemia and Blackfan Diamond Anaemia were excluded by means of Diepoxybutane (DEB) and genetic test respectively. Electroencephalogram and magnetic resonance imaging did not detect any abnormality.

## Material and methods

### Array-comparative genomic hybridization (array-CGH)

Patient’s DNA was isolated from bone marrow and buccal swab, using QIAamp DNA mini kit (Qiagen). Array-CGH was performed on both samples in accordance with manufacturer’s instructions, using Agilent Human CGH Microarray 60 K Kit (Agilent Technologies Array-CGH Kits, Santa Clara, CA). Images were obtained by Agilent scanner (G2505C) and data were extracted by Feature Extraction software (v.10.7.3.1). A graphical overview of the results was obtained using Agilent Genomic Workbench 7.0.4. Copy number variations (CNVs) were identified with the ADM2 (Aberration Detection Method) algorithm and filtered consulting the Database of Genomic Variants [http://dgv.tcag.ca/dgv/app/home] and UCSC Database [https://genome.ucsc.edu/].

### Fluorescent in situ hybridization

FISH (Fluorescent In Situ Hybridization) was performed on metaphase chromosome preparations of the patient,herparents, her brothers and her paternal grandparents.. The rearrangements revealed by array CGH were confirmed using different locus-specific BAC (Bacterial Artificial Chromosome) probes [RP11-731D23 [hg19] (93016930_93252167) at 8q21.3, RP11-4I24 [hg19](96024972_96164159) at1p21.3, RP11-545A9 [hg19] (92832705_93029934) at 1p22.1]. The BAC clones were selected from the University of California Santa Cruz (UCSC) genome browser (http://genome.ucsc.edu). FISH slides were analyzed with an Eclipse 80i (Nikon Instruments Europe B.V.), and images were captured using Genikon software (Nikon Instruments S.p.A.)

Whole exome-sequencing (WES) WES analysis was performed on patient’s DNA, isolated from bone marrow sample. Library preparation and whole exome capture was performed by using the high-throughput NimbleGen SeqCap Exome Enrichment kit (Roche https://www.roche.com/), according to the manufacture’s protocol, and sequenced on an Illumina NextSeq 550 platform. The BaseSpace pipeline (Illumina, https://basespace.illumina.com/) and the VariantStudio software (Illumina, http://variantstudio.software.illumina.com/) were used for the variant calling and annotating variants, respectively. Sequencing data were aligned to the hg19 human reference genome.

## Results

### Array-comparative genomic hybridization (array-CGH)

array CGH analysis performed on DNA from bone marrow and buccal swab detected a duplication, 4.3 Mb in size, of the short arm of chromosome 1, at 1p22.1p21.3 region, and a deletion, 2.1 Mb in size, on the long arm of chromosome 8, at 8q21.3q22.1,: arr [GRCh37]1p22.1p21.3(92122248_96403391)× 3, 8q21.3q22.1(92243681_94298184)× 1.

### FISH analysis

In order to confirm the result and to evaluate parental segregation, FISH analysis was first carried out on patient’s and her parents’ metaphases. The father presented a balanced CCR, consisting of two insertions on chromosomes 1 and 8. In detail, the 1p22.1p21 region was translocated onto the long arm of a chromosome 8, approximately at 8q12, while a different segment of the same chromosome 8, 8q21.3q22.1, was inserted onto the short arm of the rearranged chromosome 1 (1p22.1) [ish ins (1;8)(p22.1;q21.3q22.1)(RP11-4I24-,RP11-731D23+;RP11-4I24+,RP11-731D23-),ins (8;1)(q12;p22.1p21.3)(RP11-4I24+,RP11-731D23-;RP11-4I24-,RP11-731D23+)].

**Fig. 2:**
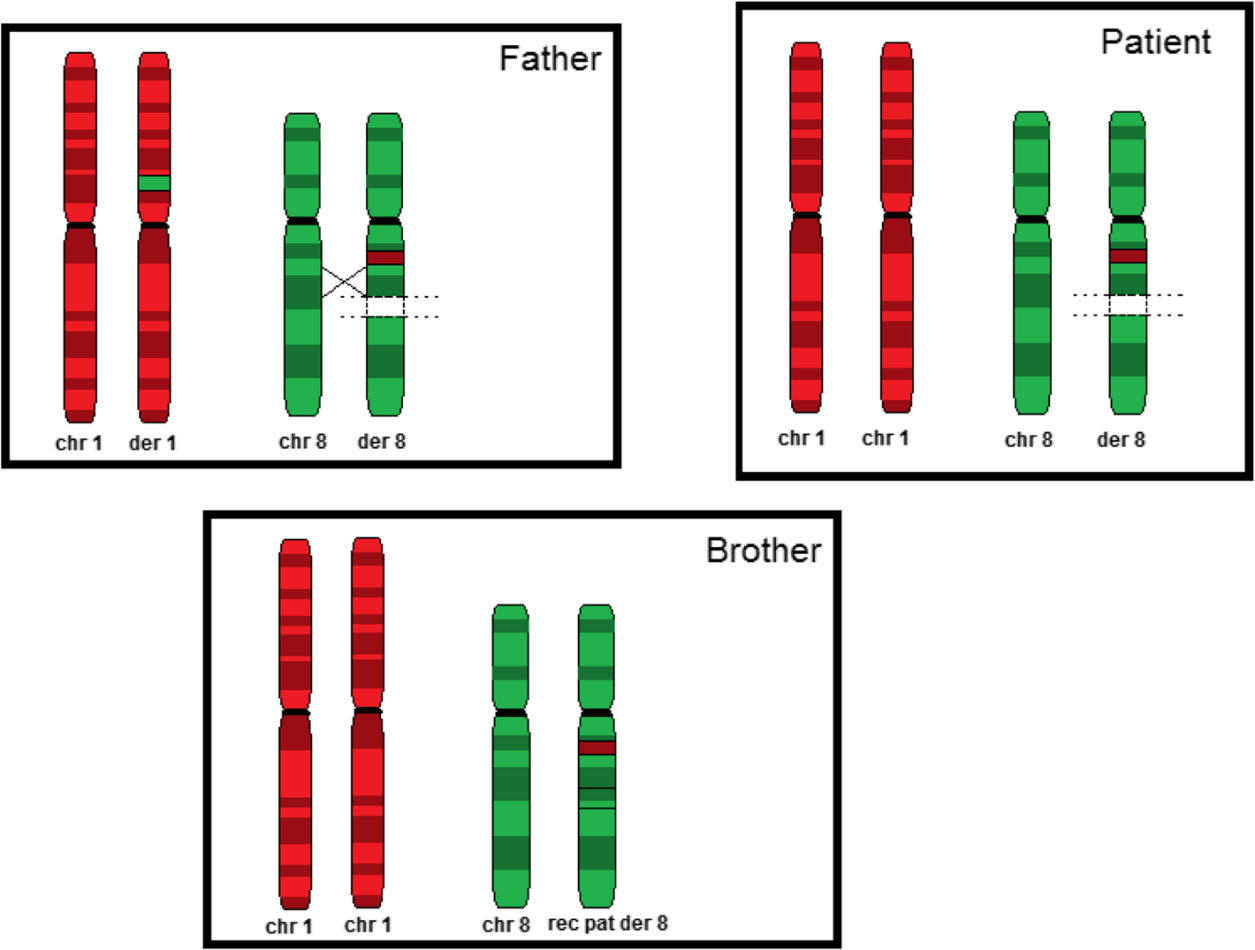
segregation analysis

The proband inherited from her father a normal chromosome 1 and the derivative chromosome 8, resulting in 1p22.1p21.3 duplication and 8q21.3q22.1 deletion (Fig. 2) [ish der(8) ins (8;1)(q12;p22.1p21.3) ins (1;8)(p22.1;q21.3q22.1) pat (RP11-4I42+,RP11-731D23-)].

**Fig. 3:**
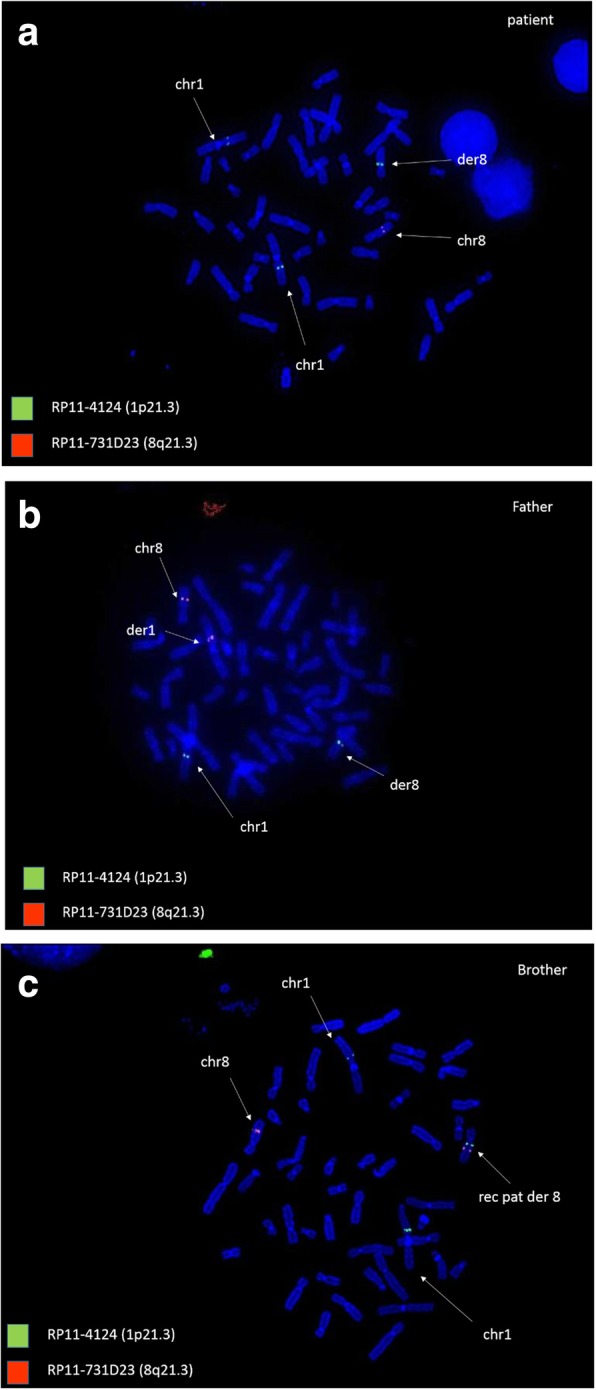
FISH analysis of metaphases from patient (**a**), her father (**b**) and her brother (**c**)

Considering the paternal origin of the der(8) chromosome, FISH analysis was then performed on the patient’s two brothers and paternal grandparents. The patient’s elder brother presented a rearranged der(8), different form the one found in his sister, harboring the sole 1p22.1p21.3 duplication [ish rec (8) ins (8;1)(q12;p22.1p21.3) pat (RP11-4I24+,RP11-731D23+)]. A crossing-over between the der(8) and the normal chromosome 8 in the paternal meiosis, had restored the missing 8q21.3q22.1 from the normal homologous to the newly derived chromosome 8 (Figs. 2 and 3). FISH analysis tested normal on the other analyzed subjects.

Whole exome-sequencing (WES) analysisAccording to myeloid phenotype of our patient, WES analysis was performed on patient’s DNA extracted from bone marrow in order to exclude a hemizygous sequence variant in RUNX1T1 gene (8q21.3) acting as a second hit. No variants were detected on RUNX1T1 coding regions, considering a mosaicism detection threshold higher than 10%.

## Discussion

We report a young girl, presenting with growth retardation, surgically resolved atrial septal defect, macrocytic anemia and mild cognitive impairment with learning disability and harboring a double genomic imbalance. The rearrangement arose from malsegregation of a 5-breakpoints paternal complex rearrangement, involving chromosome 1 and 8 (Fig. 2), which resulted in a 4.3 Mb duplication at 1p22.1p21.3 and in a 2.1 Mb deletion at 8q21.3q22.1. The 1p22.1p21.3 duplication, involved 52 UCSC genes, including 8 reported as OMIM Disease Causing (BRDT, GLML, GFI1, RPL5, GCLM, ABCA4, ABCD3, ALG14). No other overlapping cases of 1p22.1p21.3 duplications have been described in literature so far and, as this duplication is not reported as benign in CNV reference databases (DGV, Database of genomic variants), it was classified as VoUS (Variant of Unknown Significance). However, despite its extension and gene content, it is unlikely that this duplicatioin had a consistent effect on the patient’s phenotype since the 10-year old carrier brother displayed an adequate cognitive and behavioral profile. The 8q21.3q22.1 deletion involved three protein coding genes (SLC26A7, RUNX1T1, TRIQK), none of which described as OMIM Disease Causing. Several patients with deletions encompassing this region were described, mostly aiming to molecularly define the Nablus Mask-Like syndrome (NMLS), a disorder characterized by distinct facial features, including tight, glistening facial skin, blepharophimosis, ocular hypertelorism, abnormal ear architecture, sparse eyebrows and upswept frontal hairline. A smaller region of overlap among affected subject suggested a 1.84 Mb critical region at 8q22.1, from 94.43 to 96.27 Mb [6]. This region, considered necessary but not sufficient for the phenotype presentation, lies downstream our patient’s deletion, with no overlap [7].

Only four cases harboring smaller and proximal deletions have been reported far [7, 8, DECIPHER 265010, 287,719]. They presented with mild intellectual disability, learning and speech delay and, in two cases, heart defects (Table 1). Interestingly, the deletion described by Huynh et al.[8] only involved RUNX1T1, which was considered an interesting candidate gene for intellectual disability. The authors referred to a previously reported case by Zhang et al. [9], in which a balanced translocation t (5;8)(q31;q21) caused RUNX1T1 inactivation in a patient presenting with mild intellectual disability, ventricular septal defect and facial dysmorphic features. RUNX1T1 is a member of the myeloid translocation gene family. It interacts with DNA-bound transcription factors and histone-modifying enzymes, having a role in transcriptional repression. RUNX1T1 is expressed in several human tissues, in particular brain and heart, suggesting that its haploinsufficiency can be associated with mild intellectual disability and heart defects [8, 9].

**Table 1:** Patients harboring 8q21.3 proximal deletion < 4 Mb and encompassing RUNX1T1

ID	reported phenotype	array-CGH results [hg19]	size	protein coding genes
Allanson 2012, pt. 6 (DECIPHER 2399)	short stature, speech an learning delay, Intellectual disability, prominent nasal bridge, mild pulmonary valve stenosis, minor facial anomalies	8q21.3q22.1 (91,953,214-95,550,581)×1	3.4 Mb	NECAB1, C8orf88, TMEM55A, OTUD6B, LRRC69, SLC26A7, **RUNX1T1**, TRIQK, FAM92A1, RBM12B, TMEM67, PDP1, CDH17, GEM, RAD54B, FSBP, KIAA1429
Huynh 2012	mild intellectual disability, learnin disability, short stature, minor facial anomalies	8q21.3 (93,010,222-93,048,079)×1 dn	38 Kb	**RUNX1T1** partially involved
DECIPHER 265010	Intellectual disability	8q21.3q22.1 (93,045,661-93,317,115)×1 inh from a parent with a similar phenotype	271 Kb	**RUNX1T1** partially involved
DECIPHER 287719	Intellectual disability	8q21.3q22.1 (92,193,866-94,430,363)×1	2.2 Mb	LRRC69, SLC26A7, **RUNX1T1**, TRIQK
our patient	atrial septal defeact, mild short stature, intellectual disability, speech and learning delay, minor facial anomalies, severe non-haemolitic anemia, sezure episodes	8q21.3q22.1 (92,243,681-94,298,184)×1	2.1 Mb	SLC26A7, **RUNX1T1**, TRIQK

The macrocytic anemia presented in our patient has not been associated previously with 8q deletion. Valli et al. [5] reported in short the present patient focusing on RUNX1T1 as a potential causative gene for the hematological findings. In fact, t (8,21)(q22;q22) translocation, resulting in a RUNX1-RUNX1T1 chimeric gene production, is one of the most frequent chromosomal abnormalities in acute myeloid leukemia. However, no evidence for an association between RUNX1T1 deletion and a myeloid phenotype has been documented so far. Considering its role as a corepressor factor, we evaluated the possible presence of a hemizygous point mutation in bone marrow tissue, following a classical two hits model. No variant was detected by direct sequencing (WES analysis), although low-level mosaicisms cannot been excluded by molecular techniques.

The expression of this complex phenotype may also be attributed to the concomitant presence of 1p duplication as a contributory cause. However, it is not possible to exclude that the patient’s hematological disease results from an independent mechanism with unknown genetics or environmental etiological basis.

## Conclusion

We report a familial chromosome rearrangement presenting with different outcomes: a father with a five breakpoints reciprocal insertion involving chromosomes 1 and 8, and two children, a girl harboring a 1p duplication and an 8q deletion, and a boy presenting with 1p duplication. Genotype-phenotype analysis suggests RUNX1T1 as a causative gene for intellectual disability and proposes 1p22.1p21.3 duplication as a benign imbalance.

## Data Availability

Data sharing is not applicable to this article as no datasets were generated or analyzed during the current study.

## Abbreviations

ADM2: Aberration Detection Method
array-CGH: Array-Comparative Genomic Hybridization
BAC: Bacterial Artificial Chromosome
CCR: Complex chromosomal rearrangements
CNVs: Copy number variations
DEB: Diepoxybutane
DECIPHER: Database of Chromosomal Imbalance and Phenotype in Humans using Ensembl Resources
DGV: Database of genomic variants
FISH: Fluorescent In Situ Hybridization
OMIM: Online Mendelian Inheritance in Man
UCSC: University of California at Santa Cruz
UCSC: University of California Santa Cruz
VoUS: Variant of unknown significance
WES: Whole exome-sequencing
